# Biofeedback effect of hybrid assistive limb in stroke rehabilitation: A proof of concept study using functional near infrared spectroscopy

**DOI:** 10.1371/journal.pone.0191361

**Published:** 2018-01-16

**Authors:** Kazuya Saita, Takashi Morishita, Hisatomi Arima, Koichi Hyakutake, Toshiyasu Ogata, Kenji Yagi, Etsuji Shiota, Tooru Inoue

**Affiliations:** 1 Department of Neurosurgery, Faculty of Medicine, Fukuoka University, Fukuoka, Japan; 2 Department of Rehabilitation Medicine, Fukuoka University Hospital, Fukuoka, Japan; 3 Department of Preventive Medicine and Public Health, Faculty of Medicine, Fukuoka University, Fukuoka, Japan; 4 Department of Neurology, Faculty of Medicine, Fukuoka University, Fukuoka, Japan; Tokai University, JAPAN

## Abstract

**Introduction:**

Robot-assisted rehabilitation has been increasingly drawing attention in the field of neurorehabilitation. The hybrid assistive limb (HAL) is an exoskeleton robot developed based on the “interactive biofeedback” theory, and several studies have shown its efficacy for patients with stroke. We aimed to investigate the mechanisms of the facilitative effect of neurorehabilitation using a single-joint HAL (HAL-SJ) and functional near-infrared spectroscopy (fNIRS).

**Materials and methods:**

Subacute stroke patients admitted to our hospital were assessed in this study for HAL eligibility. We evaluated motor-related cortical activity using an fNIRS system at baseline and immediately after HAL-SJ treatment on the same day. Cortical activity was determined through the relative changes in the hemoglobin concentrations. For statistical analysis, we compared the number of flexion/extension movements before and immediately after HAL-SJ treatment using paired t-test. fNIRS used both the methods of statistical parametric mapping and random effect analysis.

**Results:**

We finally included 10 patients (eight men, two women; mean age: 66.8 ± 12.0 years). The mean number of flexion/extension movements within 15 s increased significantly from 4.2 ± 3.1 to 5.3 ± 4.1 immediately after training. fNIRS showed increased cortical activation in the primary motor cortex of the ipsilesional hemisphere immediately after HAL-SJ treatment compared to the baseline condition.

**Conclusions:**

This study is the first to support the concept of the biofeedback effect from the perspective of changes in cortical activity measured with an fNIRS system. The biofeedback effect of HAL immediately increased the task-related cortical activity, and this may address the functional recovery. Further studies are warranted to support our findings.

## Introduction

Stroke is a debilitating, severe disorder potentially resulting in severe impairment, and it is an important issue for patients with stroke to improve the motor function of the paretic limbs to preserve their social activity and quality of life [1]. Previous study has shown that prognosis of motor function is determined within four to five weeks after stroke [1, 2]. Hence, it is important for therapists to provide an effective treatment from the early stage after stroke onset. Robotic therapy is considered to be able to generate high intensity training and its efficacy has been investigated in the field of neurorehabilitation [3–6].

Among various types of rehabilitation robots, the hybrid assistive limb (HAL; Cyberdyne Inc., Ibaraki, Japan) is a unique wearable exoskeleton robot developed based on the "interactive biofeedback" theory [7], and several studies have shown its efficacy for stroke patients with motor disability [8–12]. The movements of the HAL robot are triggered by the bioelectrical signal (BES) detected from the muscle. HAL supports the voluntary movements of the impaired limb, and generates the sensory feedback of the successful movements to the brain [13]. The brain is, therefore, activated by the sensory feedback, and the signal of the motor network is strengthened. Thus, the HAL treatment forms a closed-loop eliciting improvement of performance and potentially promotes neuroplasticity. In subacute stroke rehabilitation, it is desirable to perform effective treatment to promote neuroplasticity in the damaged brain, and in this context HAL-assisted treatment is considered an effective approach. However, no studies have investigated the interactive biofeedback effects of rehabilitation using HAL for patients with subacute stroke.

In functional neuroimaging studies for clinical rehabilitation, non-invasive modalities such as electroencephalography (EEG), magnetoencephalography (MEG), functional magnetic resonance imaging (fMRI), and functional near infrared spectroscopy (fNIRS) have been applied [14–16]. Among these imaging modalities, fNIRS has advantage in that less physical restriction is required for the subjects compared with other modalities. Especially, the fNIRS system has been widely applied to rehabilitation studies in stroke patients [17, 18]. The fNIRS measures neuronal activities based on neurovascular coupling by detecting changes in oxygenated hemoglobin (HbO_2_) and deoxygenated hemoglobin (HHb) concentration in the target cerebral cortices [19]. This system enables the investigation of the trajectory of the chronological changes in the neural activities. We, therefore, aimed to evaluate the mechanisms of the facilitative effect of HAL-assisted rehabilitation using an fNIRS system.

## Materials and methods

### Study design

**Fig 1:**
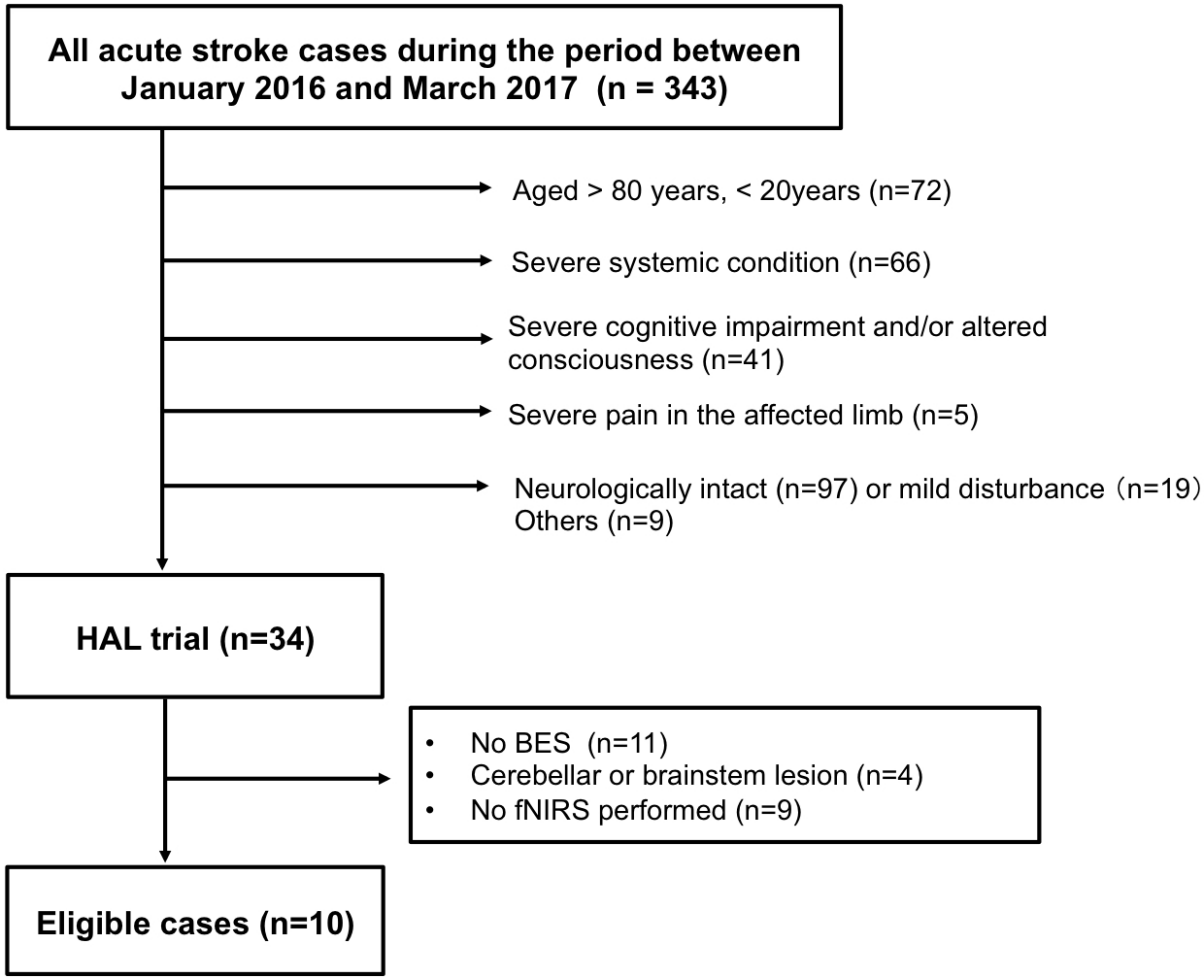
Enrollment of patients.

A total of consecutive 343 patients with subacute stroke admitted to our hospital were assessed in this study during the period between January 2016 and March 2017. We included patients between 20 and 80 years old (excluded, n = 72). We excluded patients with severe systemic conditions (n = 66), severe cognitive impairment and/or altered consciousness who were unable to follow instructions (n = 41), and severe pain in the affected limb (n = 5). In addition, we excluded patients who were neurologically intact (n = 97) or had mild disturbance (n = 19), and nine patients for miscellaneous reasons such as social issues. Finally, 10 patients were included in the study (Fig 1). As the baseline clinical evaluation, the mean National Institutes of Health Stroke Scale (NIHSS; range 0–42) score [20], Fugl-Meyer Assessment for upper extremities (FMA; range 0–66) score [21], Action Research Arm Test (ARAT; range 0–57) [22], and Mini-Mental State Examination (MMSE; range 0–30) [23] were administered. Written informed consent was obtained from each patient, and this study was conducted with approval from the institutional review board of our institution (IRB) named Fukuoka University-Medical Ethics Review Board. Our IRB approved this study protocol.

Once vital signs were stabilized after two weeks from onset, we evaluated the motor-related cortical activity of these patients using an fNIRS system under two conditions: prior to and immediately after single-joint HAL (HAL-SJ) treatment on the same day. For the task of the experiment, we instructed patients to repeat elbow flexion and extension movements without HAL-SJ.

### HAL-SJ

**Fig 2:**
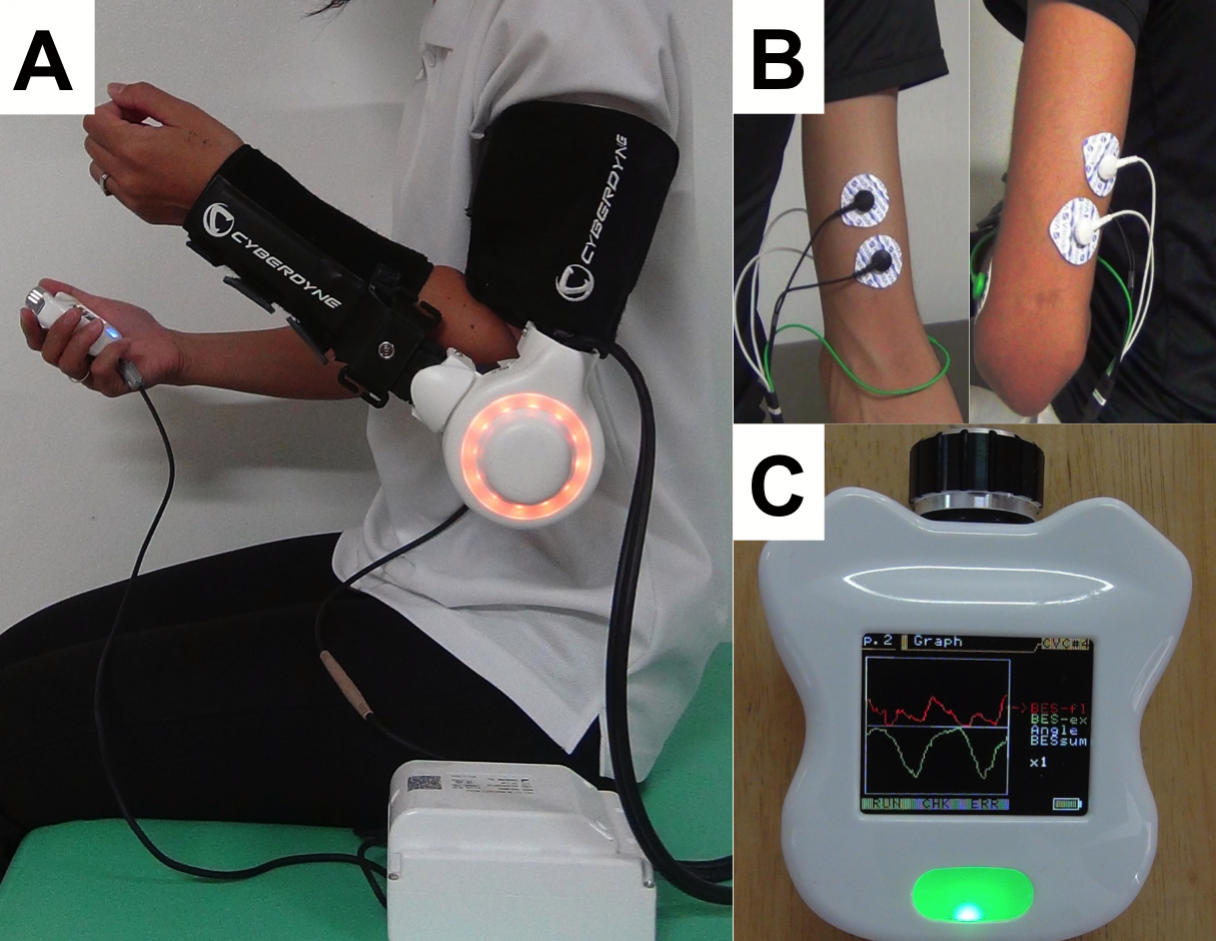
Overview of HAL-SJ. **(A) HAL-SJ attached to upper limb. (B) The location of electrode detecting BES from the biceps and triceps muscles. (C) The controller showing the BES.** Red and green waves on the monitor indicate flexor and extensor muscles, respectively.

The HAL-SJ has a power unit, and two attachments for the forearm and upper arm. This robot is small enough to be wearable as the weight is only 1.5 kg, and the range of motion of the joint is 120°. BES detected from the biceps and triceps muscles triggers the movement of HAL-SJ. A light-emitting diode (LED) is internalized in the joint of the robot suit to give visual feedback to the patient and the therapist. The LED shows different colors depending on the movement (Fig 2). Assist-gain has different levels, from 0 (no assist) to 100, to assist joint movement, and a therapist may adjust the flexion/extension balance for each training level using the controller. The controller mounts a monitor showing the BES from flexor and extensor muscles.

### NIRS study

A continuous-wave NIRS system (FOIRE-3000, Shimadzu Co., Kyoto, Japan) was used for this experiment. The infrared light wavelengths were 780, 805, and 830 nm, and the time resolution was 0.13 s. Similar to our previous studies [10, 24], we arranged the 48 channels with 32 optodes (16 emitters and 16 detectors) over the frontal and parietal areas. Based on the modified Beer-Lambert law, we acquired HbO_2_ and HHb levels by following changes in levels of cortical concentration. We used a block design for the experiment. Each cycle consisted of three periods of 15 seconds (rest–task–recovery), and the study subjects were instructed to repeat seven cycles.

Six right-handed healthy volunteers (two women and four men) were also enrolled to obtain control data to identify the location of the normal task-related cortical activation during the elbow flexion/extension movements. The mean age was 58.7±7.1. Regarding the fNIRS measurement, they carried out right elbow flexion/extension movements 15 times during 15 seconds in each task cycle, and they repeated seven task cycles as stroke subjects performed.

To visualize the fNIRS data as a t-statistical map, we used NIRS-SPM (KAIST, Daejeon, South Korea) [25], which is a MATLAB (Mathworks, Natick, MA)-based software package. With the information of non-invasive 3-D digitizer, we estimated the spatial position of the fNIRS channel locations on the Montreal Neurological Institute (MNI) coordinate system. The cerebral cortices were mapped on the basis of MNI brain coordinate and Brodmann areas (BA) (S1 Table).

### Statistical analysis

First, we compared the number of flexion/extension movements before and immediately after HAL-SJ treatment to clinically evaluate its facilitative effect. We performed paired t-tests for comparisons and p < 0.05 was set as the statistical threshold in the analysis.

To prove the facilitative effect of HAL-assisted rehabilitation for stroke cases shown by fNIRS, we used both the methods of SPM and random effect analysis to determine which channel of the fNIRS system shows statistically significant changes in the activity of the areas of interest comparing the pre- versus post-treatment conditions. For the analysis we used the NIRS-SPM software as a mass-univariate approach based on the generalized linear model (GLM) to analyze the fNIRS data. We selected the Wavelet-MDL for detruding methods and modeled the hemodynamic response function. In the group analysis, the SPM t-statistic maps were superimposed on the standardized brain according to the MNI coordinate system. HbO_2_ and HHb levels were considered significant at an uncorrected threshold of p < 0.01.

To corroborate the NIRS-SPM findings, the amplitude of the change in HbO_2_ level between the rest and on-task periods at each channel was compared before and immediately after HAL-SJ treatment using hierarchical mixed models with fixed intervention (before or immediately after HAL-SJ), fixed period (rest or on-task), and random individual effects. To control the false discovery rate (FDR) in multi-channel testing, we used the Benjamin and Hochberg methods [26]. We controlled the FDR at q-value < 0.01.

We classified 10 cases into two groups based on the amount of changes in the HbO_2_ with the task performance at the baseline fNIRS measurement: group 1 (5 cases with small cortical activity changes (< median)) and group 2 (5 cases with large cortical activity changes (≥ median)). Differences in effects of HAL training between subgroups defined by baseline value were tested by adding interaction terms to the statistical models. We used the SAS software, version 9.4 (SAS institute Inc., Cary., NC) for these statistical analyses.

## Results

**Fig 3:**
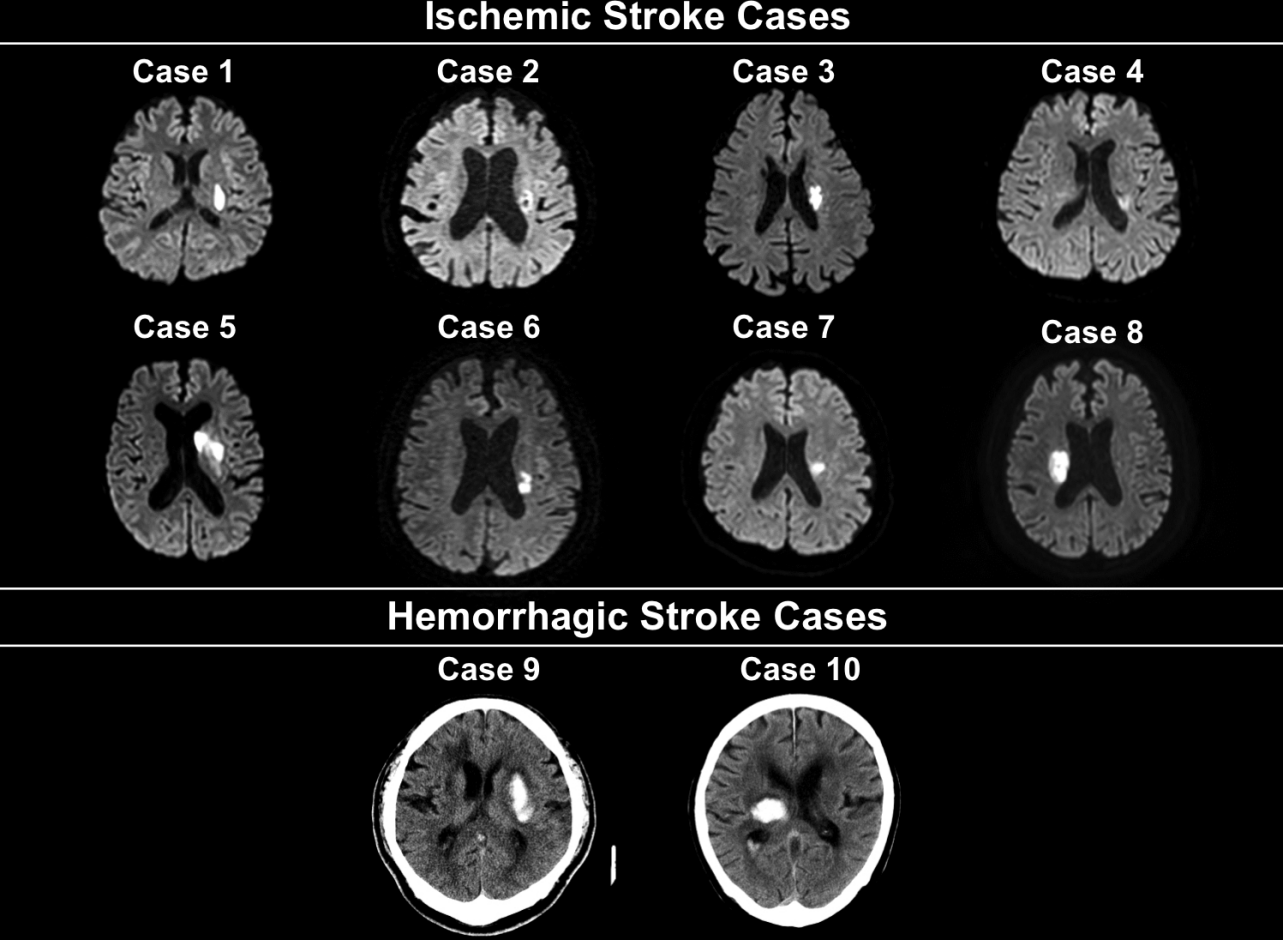
MRI/CT images presenting the stroke lesion of 10 study subjects. Diffusion-weighted images and CT images are presented to demonstrate the stroke lesions of ischemic and hemorrhagic stroke cases, respectively.

We included 10 patients (two women and eight men; mean age: 66.8 ± 12.0 years). All study participants were right handed. Of the 10 patients, eight and two had ischemic and hemorrhagic stroke, respectively. Stroke lesions were detected in the left and right hemispheres in eight and two patients, respectively. All of eight ischemic stroke cases had an ischemic lesion in the corona radiata, and one of two hemorrhagic stroke patients had a lesion in the thalamus and the other had a lesion in the putamen. Representative brain images are presented for each case in Fig 3. HAL-SJ treatment was initiated a mean 23.9 ± 15.2 days from stroke onset. The mean NIHSS score was 5.0 ± 3.0. The mean FMA and ARAT scores were 36.9 ± 24.2 and 21.5 ± 24.7, respectively. The mean MMSE score was 26.3 ± 3.0. The mean numbers of flexion and extension movements within 15 s were 4.2 ± 3.1 to 5.3 ± 4.1 at baseline and immediately after training, respectively (p < 0.05, r = 0.65). These demographics are summarized in Table 1.

**Table 1:** Patient demographics. ARAT = Action Research Arm Test, FMA = Fugl-Meyer Assessment, MMSE = Mini-Mental State Examination, NIHSS = National Institutes of Health Stroke Scale.

No.	Age	Sex	Location of lesion	Date of experiment	MMSE	NIHSS	FMA	ARAT
**Ischemic stroke cases**								
**1**	51	F	Left corona radiata	20	27	6	39	13
**2**	80	F	Left corona radiata	65	29	6	13	0
**3**	78	M	Left corona radiata	16	26	10	7	0
**4**	66	M	Left corona radiata	29	28	1	62	53
**5**	66	M	Left corona radiata	21	22	4	66	57
**6**	55	M	Left corona radiata	16	29	0	65	57
**7**	78	M	Left corona radiata	18	22	8	7	0
**8**	68	M	Right corona radiata	25	30	6	16	0
**Hemorrhagic stroke cases**								
**9**	48	M	Left putamen	14	N/A	6	47	20
**10**	78	M	Right thalamus	15	24	3	47	15
**Mean±SD**	66.8**±**12.0			23.9±15.2	26.3±3.0	5.0±3.0	36.9±24.2	21.5±24.7

**Fig 4:**
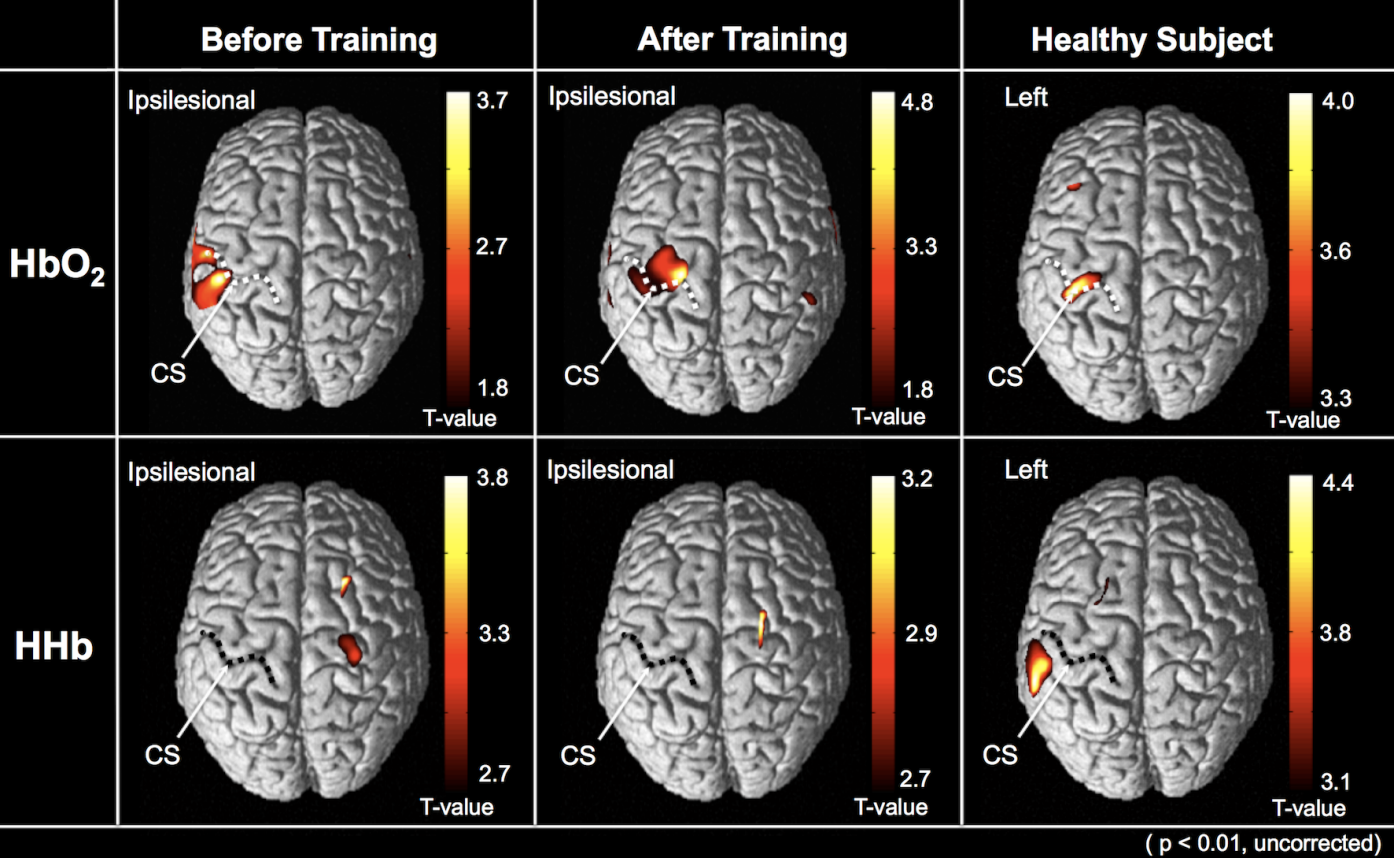
Results of group analysis for NIRS-SPM. The averaged cortical activities from all patients are depicted on the above view of the standardized brain models. Cortical activity was increased immediately after training on the same day. The upper and lower represents the cortical activation in HbO_2_ and HHb level, respectively. Each performing status was also significantly improved compared to other regions (uncorrected, p < 0.01). Dotted lines indicate central sulcus (CS) on the normalized brain images.

On the t-statistical map of the NIRS-SPM group analysis, the HbO_2_ level changes in the hand bump area of the ipsilesional primary motor cortex (M1) were not significant compared to those in other cortical areas prior to treatment; however, the same area showed significant increase of the activation level after treatment (uncorrected, p < 0.01) (Fig 4). However, the HHb level changes were not significant in either condition.

The NIRS-SPM group analysis of healthy volunteers showed that the cortical activity during the task was increased in the left sensorimotor cortex as shown by HbO_2_ data (uncorrected, p < 0.01), and this finding was similar to the status of post-HAL training in the study subjects.

**Fig 5:**
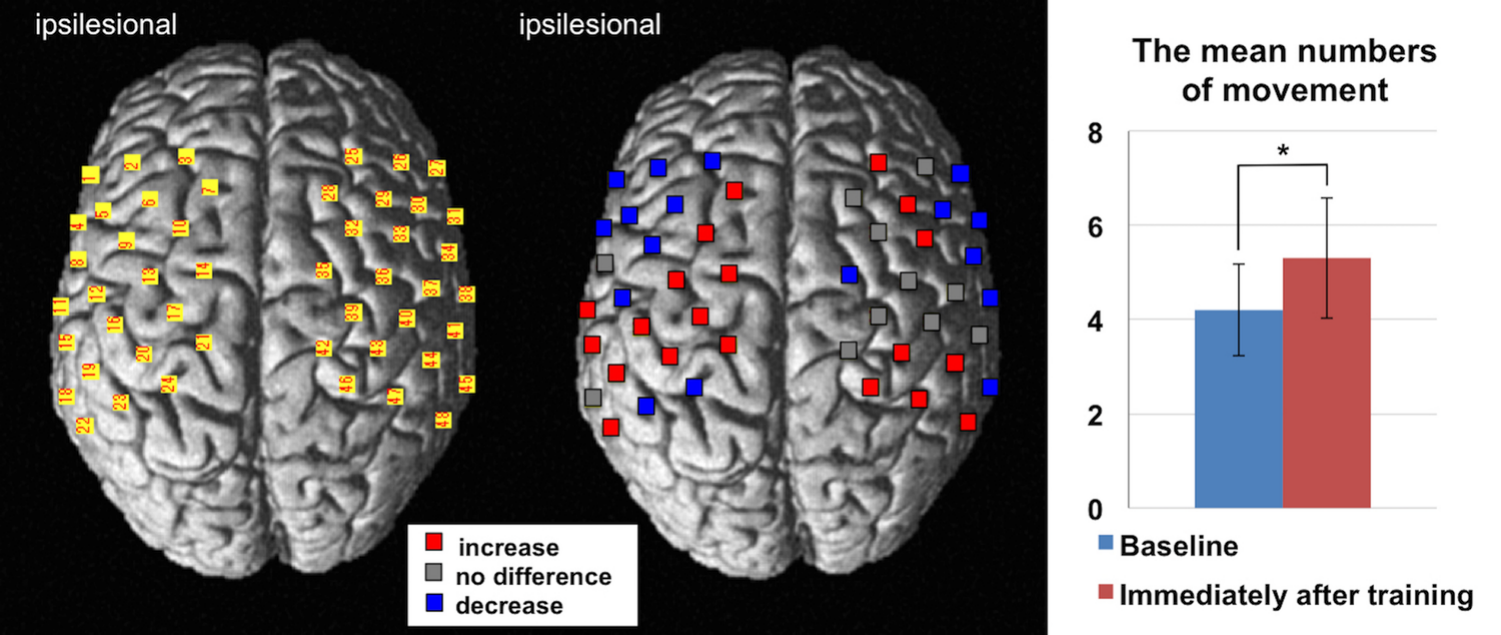
Results of group analysis comparing pre-versus post-HAL treatment. The cortical activity of change represents the comparing pre-and post-HAL treatments. For the left image, the numbers of NIRS channels were superimposed on the standardized brain according to the MNI coordinate system. For the right image, red and blue indicate increase and decrease in the HbO_2_ level, respectively (FDR corrected, p < 0.01). Gray indicates that the channels were not significantly changed following HAL-assisted rehabilitation. The mean number of flexion/extension movements within 15 seconds is indicated as a graph on the right of the figure. Motor performance was statistically significantly improved compared to the baseline status. * p-value was set 0.05.

The random effect analysis revealed that the amplitude of increase in the HbO_2_ levels was more pronounced immediately after HAL treatment especially in the motor areas of the lesioned hemisphere (S2 Table). Among all channels, the Ch17 (corresponding to the premotor cortex and M1 of ipsilesional hemisphere) was the most increased immediately after training compared to the baseline (ΔHbO_2_ (difference in change) = 0.0128, confidence interval (CI): 0.0117 to 0.0139, p < 0.0001). Fig 5 shows the changes in the amplitude of the HbO_2_ levels during the task at each channel.

A sub-analysis concerning four regions of interest (channels 13, 16, 17, and 20 located over hand/arm area of the ipsilesional M1) showed there are significant differences between group 1 and group 2 (Table 2). This result implicates that cases with larger increase in HbO_2_ levels at baseline with motor tasks are likely to respond to HAL-assisted rehabilitation.

**Table 2:** Comparison between Group 1 (smaller changes) and Group 2 (larger changes) based on the amount of changes in the HbO_2_ during task performance at the baseline fNIRS evaluation CI = Confidence Interval.

		Difference in change (95% CI)	P interaction
**Ch13**	Group 1	-0.0027 (-0.0044 to -0.0010)	< 0.001
	Group 2	0.0103 (0.0093 to 0.0133)	
**Ch16**	Group 1	-0.0002 (-0.0015 to 0.0011)	< 0.001
	Group 2	0.0136 (0.0125 to 0.0147)	
**Ch17**	Group 1	0.0057 (0.0044 to 0.0070)	< 0.001
	Group 2	0.0204 (0.0190 to 0.0218)	
**Ch20**	Group 1	0.0094 (0.0076 to 0.0110)	0.0220
	Group 2	0.0071 (0.0061 to 0.0081)	

## Discussion

Our study possibly demonstrated that HAL-assisted rehabilitation immediately induced task-related neuroplasticity for upper limb motor function in patients with stroke. In addition, the two different statistical approaches in this study both showed more activation of the motor cortex in the lesioned hemisphere. The present fNIRS finding may implicate that HAL treatment enhanced cortical activity in the ipsilesional M1 in response to significant improvement of performance status in patients with subacute stroke. Our previous study of the acute stroke cases suggested that the HAL-SJ might have accelerated the functional recovery compared with conventional rehabilitation [9], and the present study may indicate the possible mechanism.

A recent study addressed the importance of motor skill learning by subsequent practice and acquisition of new motor skills in the ipsilesional M1 [27]. Several studies have shown the efficacy of neurorehabilitation programs addressing use-dependent neuroplasticity, such as constraint-induced movement therapy (CIMT) and robot rehabilitation [28–31]. Repetitive training eliciting voluntary movement has been considered to produce more effective functional recovery than passive exercise training [32]. In this context, HAL-assisted treatment may facilitate neuroplasticity associated with motor skill learning in patients with stroke in the early stage.

**Fig 6:**
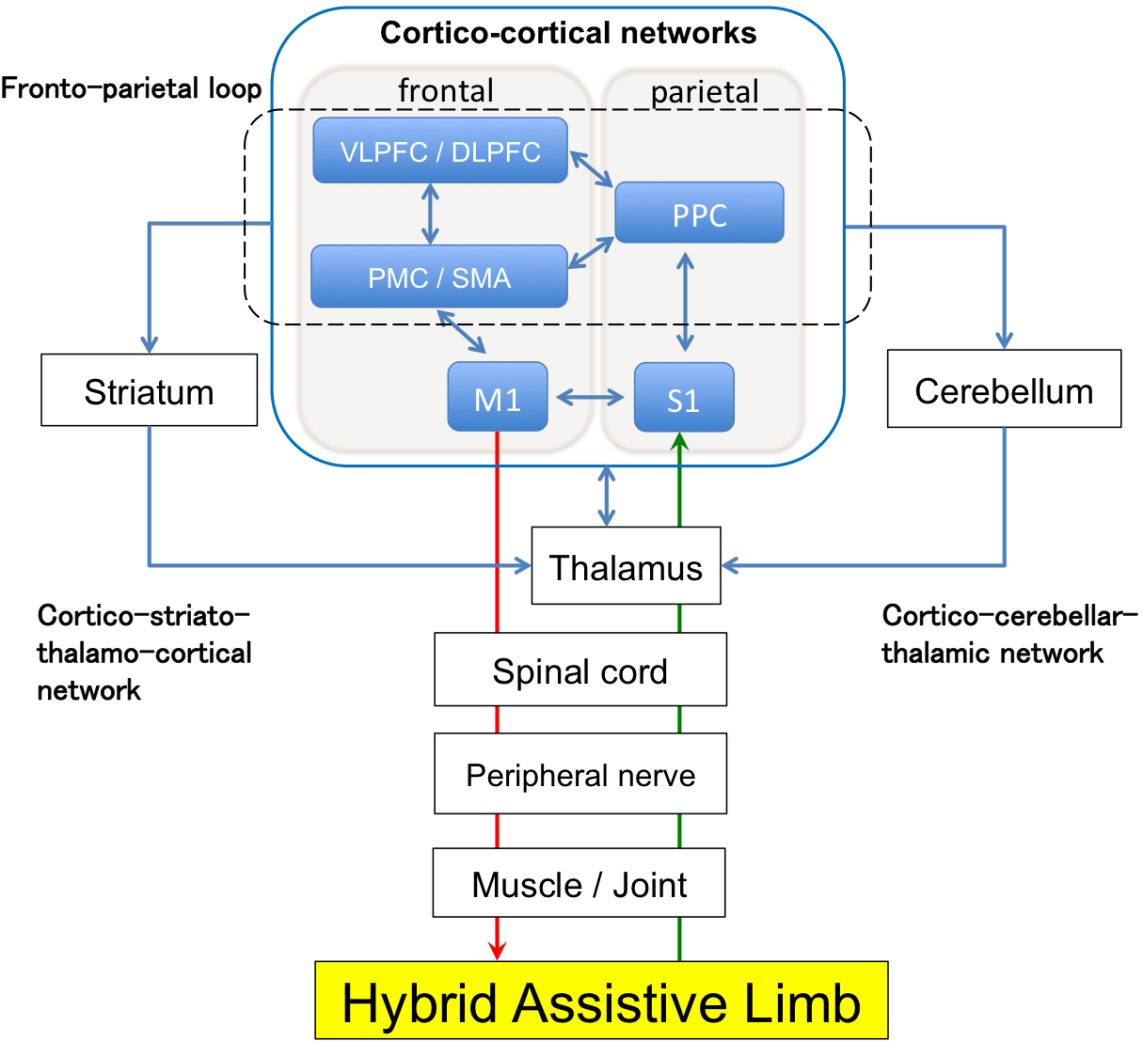
A proposed biofeedback model of HAL treatment.

Concerning the mechanisms of HAL treatment, the robot assists the voluntary movements generating a strong proprioceptive sensation, which is sent back to the brain to generate stronger motor output signals [7]. In this process, we speculate that the sensory signals pass through the thalamus to the fronto-parietal networks, involving the primary sensory cortex, and the network connections are strengthened to exert a robust signal from the motor cortex down to the muscle. We also consider that the basal ganglia and the cerebellar circuits play a crucial role in the process of motor skill learning using HAL. Here, we propose a brain network model potentially activated by the biofeedback effect of HAL rehabilitation (Fig 6). Therapists and clinicians, however, should be aware that HAL therapy requires BES. As previous studies have shown that functional recovery was correlated to corticospinal tract (CST) integrity [28, 33], the clinical effect of HAL therapy may depend on CST integrity. This point should be addressed by future studies.

There are several important limitations in this study. It included a small sample size and there was no control. As we did not include a follow-up, this study does not demonstrate the efficacy of HAL treatment for stroke survivors. It should be mentioned that the evaluation timings ranged from 14 to 65 days from the stroke onset, and this issue may have affected the results as previous fMRI studies showed that the amount of the ipsilesional cortical activity is reportedly different between acute and chronic stages [34]. Additionally, even though results of this study positively support the biofeedback effect of the HAL, we are unable to exclude the influence of the learning by repetition as the control subjects were not evaluated in this study. Furthermore, as a limitation of the imaging analysis, fNIRS is unable to measure the activities of deep brain areas, such as the cerebellum and basal ganglia. Although the HAL is a well-manufactured robot, it is still in development, and the potential of HAL-assisted rehabilitation depends on robot engineering technology. Our study showed the current status of HAL-assisted rehabilitation, and we consider the future development of the robot will address the remaining issues.

## Conclusions

This study is the first to support the concept of the interactive biofeedback effect from the perspective of changes in cortical activity measured with an fNIRS system. The biofeedback effect of HAL immediately increased the task-related cortical activity, and this may address the functional recovery. Further studies are warranted to corroborate our findings.

## Supporting information

S1 Table Cortical mapping based on the MNI coordination and brodmann area. DLPFC: Dorsal lateral prefrontal cortex, VLPFC: Ventral lateral prefrontal cortex, M1: primary motor cortex, PMC: premotor cortex, PPC: posterior parietal cortex, S1: primary somatosensory cortex, SM1: primary sensorimotor cortex, SMA: supplemental motor area.
(DOCX)

S2 Table HbO_2_ level for fNIRS. Values are mean (95% confidence interval).
(DOCX)
