# Supplementary material for: Biofeedback effect of hybrid assistive limb in stroke rehabilitation: A proof of concept study using functional near infrared spectroscopy
Source: PLoS One. 2018 Jan 16;13(1):e0191361. doi: 10.1371/journal.pone.0191361 (PMC5770063; doi:10.1371/journal.pone.0191361)
Supplement: S1 Table — DLPFC: Dorsal lateral prefrontal cortex, VLPFC: Ventral lateral prefrontal cortex, M1: primary motor cortex, PMC: premotor cortex, PPC: posterior parietal cortex, S1: primary somatosensory cortex, SM1: primary sensorimotor cortex, SMA: supplemental motor area. (DOCX) [file pone.0191361.s001.docx]

**S1 Table: Cortical mapping based on the MNI coordinate system and Brodmann area**

| **Ipsilesional hemisphere** | | **Contralesional hemisphere** | | **Brodmann area** | |
| --- | --- | --- | --- | --- | --- |
| **Ch** | **MNI coordinate**  **(x / y / z)** | **Ch** | **MNI coordinate**  **(x / y / z)** | **Area No.** | **Estimated anatomical label** |
| **1** | -56.3 / 26.4 / 23.2 | **27** | 59.2 / 27.2 / 21.3 | 44, 45 | VLPFC |
| **2** | -42.8 / 29.6 / 44.4 | **26** | 46.8 / 31.1 / 42.3 | 9, 44, 45, 46 | DLPFC / VLPFC |
| **3** | -25.2 / 32.2 / 56.3 | **25** | 29.8 / 32.3 / 54.9 | 8, 9 | DLPFC |
| **4** | -62.4 /10.2 / 19.2 | **31** | 64.6 / 11.4 / 18.0 | 6, 44, 45 | PMC / VLPFC |
| **5** | -51.8 / 15.2 / 43.0 | **30** | 53.8 / 16.7 / 41.6 | 9, 44 | DLPFC / VLPFC |
| **6** | -37.1 / 18.8 / 59.2 | **29** | 40.1 / 20.3 / 57.7 | 8, 9 | DLPFC |
| **7** | -17.6 / 21.2 / 66.6 | **28** | 20.8 / 21.1 / 66.1 | 8 | DLPFC |
| **8** | -60.3 / 0.1 / 39.5 | **34** | 62.4 / 2.6 / 39.4 | 6 | PMC |
| **9** | -45.1 / 5.0 / 57.8 | **33** | 46.7 / 6.5 / 57.3 | 6 | PMC |
| **10** | -27.6 / 8.3 / 67.7 | **32** | 28.7 / 8.6 / 67.2 | 6 | PMC |
| **11** | -67.1 / -15.2 / 30.8 | **38** | 69.0 / -12.8 / 31.8 | 1, 2 | S1 |
| **12** | -55.0 / -11.6 / 53.6 | **37** | 55.9 / -9.0 / 54.4 | 4, 6 | M1 / PMC |
| **13** | -37.4 / -7.5 / 67.4 | **36** | 38.7 / -6.8 / 67.2 | 6 | PMC |
| **14** | -18.3 / -4.8 / 75.0 | **35** | 18.9 / -3.9 / 75.0 | 6 | PMC (SMA) |
| **15** | -63.1 / -26.7 / 46.3 | **41** | 63.9 / -23.9 / 49.3 | 1, 2, 3, 4 | SM1 |
| **16** | -47.0 / -22.5 / 65.1 | **40** | 47.0 / -20.2 / 65.9 | 3, 4 | SM1 |
| **17** | -29.3 / -18.3 / 74.1 | **39** | 29.3 / -17.8 / 74.4 | 4, 6 | PMC (SMA) / M1 |
| **18** | -66.6 / -43.1 / 32.1 | **45** | 67.7 / -40.9 / 35.3 | 40 | PPC |
| **19** | -54.9 / -37.0 / 56.0 | **44** | 55.2 / -34.2 / 57.8 | 1, 2, 40 | S1, PPC |
| **20** | -38.9 / -31.7 / 70.4 | **43** | 38.1 / -29.5 / 71.5 | 3, 4 | SM1 |
| **21** | -19.4 / -28.2 / 76.9 | **42** | 19.0 / -28.9 / 78.2 | 4 | SM1 |
| **22** | -59.6 / -53.7 / 44.1 | **48** | 60.1 / -51.7 / 47.5 | 40 | PPC |
| **23** | -45.1 / -46.0 / 62.2 | **47** | 44.4 / -43.4 / 64.7 | 40 | PPC |
| **24** | -28.8 / -41.8 / 73.8 | **46** | 27.4 / -40.0 / 74.9 | 1, 2, 5, 7 | S1 / PPC |

DLPFC: Dorsal lateral prefrontal cortex, VLPFC: Ventral lateral prefrontal cortex, M1: primary motor cortex, PMC: premotor cortex, PPC: posterior parietal cortex, S1: primary somatosensory cortex, SM1: primary sensorimotor cortex, SMA: supplemental motor area
