# Supplementary material for: Biofeedback effect of hybrid assistive limb in stroke rehabilitation: A proof of concept study using functional near infrared spectroscopy
Source: PLoS One. 2018 Jan 16;13(1):e0191361. doi: 10.1371/journal.pone.0191361 (PMC5770063; doi:10.1371/journal.pone.0191361)
Supplement: S2 Table — Values are mean (95% confidence interval). (DOCX) [file pone.0191361.s002.docx]

**S2 Table: HbO_2_ level for fNIRS**

| **Ch** | **Before HAL-SJ training** | | | **Immediately after HAL-SJ training** | | | **Difference in change**  **(95% CI)** | **P-value** | **q-value** |
| --- | --- | --- | --- | --- | --- | --- | --- | --- | --- |
|  | **Rest**  **(95% CI)** | **On task**  **(95% CI)** | **Change**  **(95% CI)** | **Rest**  **(95% CI)** | **On task**  **(95% CI)** | **Change**  **(95% CI)** |  |  |  |
| **1** | -0.0097  (-0.0412 to 0.0219) | -0.0058  (-0.0373 to 0.0257) | 0.0039  (0.0031 to 0.0047) | 0.0073  (-0.0071 to 0.0217) | 0.0094  (-0.0050 to 0.0237) | 0.0020  (0.0014 to 0.0027) | -0.0019  (-0.0029 to -0.0009) | .0074 | .0096 |
| **2** | 0.0007  (-0.0194 to 0.0207) | -0.0003  (-0.0204 to 0.0197) | -0.0010  (-0.0018 to -0.0003) | -0.0018  (-0.0197 to 0.0161) | -0.0057  (-0.0236 to 0.0122) | -0.0039  (-0.0045 to -0.0033) | -0.0029  (-0.0039 to -0.0019) | < .0001 | < .0001 |
| **3** | -0.0012  (-0.0215 to 0.0191) | -0.0020  (-0.0223 to 0.0183) | -0.0008  (-0.0015 to -0.0001) | -0.0052  (-0.0255 to 0.0151) | -0.0121  (-0.0324 to 0.0082) | -0.0069  (-0.0076 to -0.0062) | -0.0061  (-0.0071 to -0.0051) | < .0001 | < .0001 |
| **4** | -0.0033  (-0.0189 to 0.0124) | 0.0020  (-0.0136 to 0.0176) | 0.0053  (0.0047 to 0.0058) | 0.0165  (0.0069 to 0.0262) | 0.0202  (0.0106 to 0.0298) | 0.0037  (0.0030 to 0.0044) | -0.0016  (-0.0024 to -0.0008) | .0034 | .0047 |
| **5** | 0.0024  (-0.0160 to 0.0207) | 0.0060  (-0.0124 to 0.0244) | 0.0036  (-0.0030 to 0.0043) | 0.0032  (-0.0093 to 0.0157) | 0.0046  (-0.0079 to 0.0171) | 0.0015  (0.0008 to 0.0021) | -0.0021  (-0.0029 to -0.0013) | < .0001 | < .0001 |
| **6** | -0.0037  (-0.0161 to 0.0086) | -0.0030  (-0.0153 to ) | 0.0007  (0.0003 to 0.0012) | -0.0053  (-0.0167 to 0.0062) | -0.0108  (-0.0223 to 0.0007) | -0.0055  (-0.0063 to -0.0047) | -0.0062  (-0.0071 to -0.0053) | < .0001 | < .0001 |
| **7** | 0.0174  (-0.0035 to 0.0382) | 0.0117  (-0.0091 to 0.0325) | -0.0057  (-0.0067 to -0.0046) | 0.0121  (-0.0005 to 0.0247) | 0.0110  (-0.0017 to 0.0236) | -0.0012  (-0.0019 to -0.0005) | 0.0045  (0.0032 to 0.0058) | < .0001 | < .0001 |
| **8** | 0.0028  (-0.0066 to 0.0121) | 0.0069  (-0.0025 to 0.0162) | 0.0041  (0.0035 to 0.0047) | 0.0010  (-0.0113 to 0.0133) | 0.0046  (-0.0077 to 0.0170) | 0.0037  (0.0031 to 0.0043) | -0.0004  (-0.0012 to 0.0004) | .3469 | .3799 |
| **9** | -0.0086  (-0.0260 to 0.0089) | -0.0062  (-0.0236 to 0.0112) | 0.0024  (0.0018 to 0.0029) | -0.0099  (-0.0293 to 0.0096) | -0.0135  (-0.0330 to 0.0059) | -0.0037  (-0.0043 to -0.0030) | -0.0061  (-0.0069 to -0.0053) | < .0001 | < .0001 |
| **10** | 0.0153  (-0.0026 to 0.0331) | 0.0097  (-0.0081 to 0.0275) | -0.0055  (-0.0065 to -0.0046) | -0.0041  (-0.0179 to 0.0097) | -0.0051  (-0.0189 to 0.0087) | -0.0010  (-0.0017 to -0.0003) | 0.0045  (0.0032 to 0.0058) | < .0001 | < .0001 |
| **11** | 0.0006  (-0.0188 to 0.0199) | 0.009  (-0.0104 to 0.0283) | 0.0084  (0.0077 to 0.0091) | 0.0034  (-0.0158 to 0.0226) | 0.0157  (-0.0035 to 0.0348) | 0.0123  (0.0115 to 0.0130) | 0.0039  (0.0029 to 0.0049) | < .0001 | < .0001 |
| **12** | 0.0026  (-0.0072 to 0.0124) | 0.0063  (-0.0035 to 0.0161) | 0.0037  (0.0032 to 0.0042) | -0.0024  (-0.0188 to 0.0140) | -0.0013  (-0.0177 to 0.0152) | 0.0012  (0.0006 to 0.0018) | -0.0025  (-0.0033 to -0.0017) | < .0001 | < .0001 |
| **13** | 0.0111  (-0.0143 to 0.0366) | 0.0169  (-0.0086 to 0.0423) | 0.0057  (0.0049 to 0.0066) | -0.0065  (-0.0298 to 0.0168) | 0.0031  (-0.0202 to 0.0264) | 0.0096  (0.0088 to 0.0104) | 0.0039  (0.0028 to 0.0050) | < .0001 | < .0001 |
| **14** | 0.0168  (0.0001 to 0.0335) | 0.0104  (-0.0063 to 0.0270) | -0.0064  (-0.0074 to -0.0054) | -0.0034  (-0.0216 to 0.0148) | -0.0047  (-0.0229 to 0.0135) | -0.0013  (-0.0022 to -0.0004) | 0.0051  (0.0037 to 0.0065) | < .0001 | < .0001 |
| **15** | -0.0017  (-0.0149 to 0.0114) | 0.0018  (-0.0113 to 0.0150) | 0.0036  (0.0029 to 0.0043 | -0.0051  (-0.0197 to 0.0096) | 0.0014  (-0.0132 to 0.0161) | 0.0065  (0.0059 to 0.0072) | 0.0029  (0.0019 to 0.0039) | < .0001 | < .0001 |
| **16** | 0.0130  (-0.0068 to 0.3280) | 0.0179  (-0.0019 to 0.3775) | 0.0049  (0.0043 to 0.0056) | -0.0043  (-0.0191 to 0.0104) | 0.0072  (-0.0075 to 0.2202) | 0.0116  (0.0109 to 0.0122) | 0.0067  (0.0059 to 0.0075) | < .0001 | < .0001 |
| **17** | 0.00157  (-0.0143 to 0.0175) | 0.0060  (-0.0099 to 0.0219) | 0.0044  (0.0037 to 0.0052) | -0.0053  (-0.0210 to 0.0103) | 0.0118  (-0.0038 to 0.0275) | 0.0172  (0.0165 to 0.0179) | 0.0128  (0.0117 to 0.0139) | < .0001 | < .0001 |
| **18** | -0.0021  (-0.0161 to 0.0119) | 0.0099  (-0.0041 to 0.0239) | 0.0120  (0.0112 to 0.0128) | -0.0087  (-0.0321 to 0.0147) | 0.0026  (-0.0209 to 0.0260) | 0.0113  (0.0104 to 0.0121) | -0.0007  (-0.0018 to 0.0004) | .2523 | .2818 |
| **19** | 0.0010  (-0.0123 to 0.0104) | 0.0022  (-0.0092 to 0.0135) | 0.0032  (0.0026 to 0.0037) | -0.0002  (-0.0189 to 0.0185) | 0.0091  (-0.0096 to 0.2785) | 0.0094  (0.0085 to 0.0102) | 0.0062  (0.0052 to 0.0072) | < .0001 | < .0001 |
| **20** | 0.0074  (-0.0075 to 0.0222) | 0.0130  (-0.0019 to 0.0278) | 0.0056  (0.0050 to 0.0062) | -0.0008  (-0.0233 to 0.0218) | 0.0128  (-0.0098 to 0.0353) | 0.0136  (0.0128 to 0.0143) | 0.0080  (0.0070 to 0.0090) | < .0001 | < .0001 |
| **21** | 0.0040  (-0.0085 to 0.0166) | 0.0067  (-0.0058 to 0.0193) | 0.0027  (0.0022 to 0.0032) | -0.0064  (-0.0202 to 0.0074) | 0.0025  (-0.0113 to 0.0163) | 0.0089  (0.0083 to 0.0095) | 0.0062  (0.0053 to 0.0070) | < .0001 | < .0001 |
| **22** | -0.0052  (-0.0390 to 0.0286) | 0.0054  (-0.0284 to 0.0393) | 0.0107  (0.0097 to 0.0117) | -0.0037  (-0.0213 to 0.0138) | 0.0103  (-0.0073 to 0.0278) | 0.0140  (0.0132 to 0.0148) | 0.0033  (0.0020 to 0.0046) | .0002 | .0002 |
| **23** | -0.0122  (-0.0389 to 0.0145) | -0.0090  (-0.0357 to 0.0178) | 0.0032  (0.0025 to 0.0040) | -0.0122  (-0.0239 to -0.0006) | -0.0113  (-0.0230 to 0.0003) | 0.0009  (0.0004 to 0.0014) | -0.0023  (-0.0033 to -0.0013) | .0003 | .0005 |
| **24** | 0.0026  (-0.0124 to 0.0177) | 0.0079  (-0.0071 to 0.0229) | 0.0053  (0.0047 to 0.0058) | -0.0150  (-0.0241 to -0.0059) | -0.0112  (-0.0204 to -0.0021) | 0.0038  (0.0033 to 0.0043) | -0.0015  (-0.0023 to -0.0007) | .0065 | .0087 |
| **25** | 0,0047  (-0.0065 to 0.0160) | 0.0096  (-0.0016 to 0.0208) | 0.0048  (0.0044 to 0.0053) | -0.0002  (-0.0214 to 0.0209) | 0.00638  (-0.0148 to 0.0275) | 0.0066  (0.0059 to 0.0073) | 0.0018  (0.0009 to 0.0027) | .0022 | .0032 |
| **26** | -0.0082  (-0.0184 to 0.0021) | -0.0057  (-0.0160 to 0.0045) | 0.0025  (0.0021 to 0.0028) | -0.0030  (-0.0140 to 0.0079) | -0.0011  (-0.0120 to 0.0099) | 0.0020  (0.0014 to 0.0025) | -0.0005  (-0.0012 to 0.0002) | .2417 | .2764 |
| **27** | -0.0042  (-0.0140 to 0.0056) | 0.0009  (-0.0089 to 0.0107) | 0.0051  (0.0046 to 0.0056) | 0.0015  (-0.0087 to 0.0117) | 0.0040  (-0.0062 to 0.0142) | 0.0025  (0.0019 to 0.0031) | -0.0026  (-0.0033 to -0.0019) | < .0001 | < .0001 |
| **28** | -0.0064  (-0.0207 to 0.0080) | -0.0043  (-0.0186 to 0.0101) | 0.0021  (0.0016 to 0.0026) | -0.0011  (-0.0155 to 0.0133) | 0.0002  (-0.0142 to 0.0147) | 0.0014  (0.0006 to 0.0021) | -0.0007  (-0.0017 to 0.0003) | .1628 | .1904 |
| **29** | -0.0112  (-0.0224 to 0) | -0.0099  (-0.0211 to 0.0013) | 0.0013  (0.0008 to 0.0019) | -0.0004  (-0.0127 to 0.0120) | 0.0035  (-0.0088 to 0.0159) | 0.0039  (0.0032 to 0.0046) | 0.0026  (0.0016 to 0.0036) | < .0001 | < .0001 |
| **30** | -0.0046  (-0.0210 to 0.0118) | -0.0029  (-0.0193 to 0.0135) | 0.0016  (0.0010 to 0.0023) | -0.0048  (-0.0148 to 0.0053) | -0.0077  (-0.0177 to 0.0024) | -0.0029  (-0.0035 to -0.0023) | -0.0045  (-0.0055 to -0.0035) | < .0001 | < .0001 |
| **31** | 0.0082  (-0.0068 to 0.0233) | 0.0206  (0.0056 to 0.0357) | 0.0124  (0.0118 to 0.0130) | 0.0064  (-0.0088 to 0.0216) | 0.0151  (-0.0001 to 0.0302) | 0.0086  (0.0079 to 0.0094) | -0.0038  (-0.0048 to -0.0028) | < .0001 | < .0001 |
| **32** | -0.0121  (-0.0193 to -0.0049) | -0.0098  (-0.0170 to -0.0025) | 0.0023  (0.0018 to 0.0028) | 0.0039  (-0.0126 to 0.0203) | 0.0076  (-0.0088 to 0.0240) | 0.0038  (0.0030 to 0.0045) | 0.0015  (0.0005 to 0.0025) | .0098 | .0124 |
| **33** | -0.0094  (-0.0207 to 0.0018) | -0.0069  (-0.0181 to 0.0043) | 0.0025  (0.0019 to 0.0032) | 0.0016  (-0.0148 to 0.0180) | 0.0063  (-0.0101 to 0.0227) | 0.0048  (0.0041 to 0.0054) | 0.0023  (0.0015 to 0.0031) | < .0001 | < .0001 |
| **34** | -0.0049  (-0.0171 to 0.0073) | -0.0019  (-0.0140 to 0.0103) | 0.0030  (0.0025 to 0.0035) | 0.0009  (-0.0098 to 0.0116) | 0.0011  (-0.0096 to 0.0118) | 0.0002  (-0.0004 to 0.0008) | -0.0028  (-0.0036 to -0.002) | < .0001 | < .0001 |
| **35** | -0.0092  (-0.0287 to 0.0104) | -0.0092  (-0.0287 to 0.0103) | 0  (-0.0009 to 0.0008) | -0.0016  (-0.0165 to 0.0133) | -0.0072  (-0.0221 to 0.0076) | -0.0057  (-0.0066 to -0.0048) | -0.0057  (-0.007 to -0.0044) | < .0001 | < .0001 |
| **36** | -0.0129  (-0.0326 to 0.0068) | -0.0091  (-0.0288 to 0.0107) | 0.0038  (0.0031 to 0.0046) | -0.0005  (-0.0204 to 0.0194) | 0.0031  (-0.0168 to 0.0230) | 0.0036  (0.0028 to 0.0044) | -0.0002  (-0.0013 to 0.0009) | .7746 | .7938 |
| **37** | -0.0122  (-0.0330 to 0.0087) | -0.0062  (-0.0270 to 0.0146) | 0.0060  (0.0050 to 0.0044) | -0.0003  (-0.0220 to 0.0214) | 0.0041  (-0.0176 to 0.0258) | 0.0044  (0.0038 to 0.0050) | -0.0016  (-0.0027 to -0.0005) | .0678 | .0811 |
| **38** | -0.0278  (-0.0597 to 0.0043) | -0.0181  (-0.0501 to 0.0139) | 0.0096  (0.0086 to 0.0107) | -0.0062  (-0.0230 to 0.0105) | -0.0003  (-0.0171 to 0.0164) | 0.0059  (0.0052 to 0.0066) | -0.0037  (-0.005 to -0.0024) | < .0001 | < .0001 |
| **39** | -0.0189  (-0.0391 to 0.0013) | -0.0159  (-0.0361 to 0.0043) | 0.0030  (0.0021 to 0.0039) | -0.0052  (-0.0203 to 0.0099) | -0.0019  (-0.0169 to 0.0132) | 0.0033  (0.0026 to 0.0041) | 0.0003  (-0.0008 to 0.0014) | .5486 | .5851 |
| **40** | -0.0216  (-0.0663 to 0.0231) | -0.0201  (-0.0649 to 0.0246) | 0.0015  (0.0001 to 0.0029) | 0.0057  (-0.0228 to 0.0342) | 0.0111  (-0.0174 to 0.0396) | 0.0054  (0.0045 to 0.0064) | 0.0039  (0.0022 to 0.0056) | .0111 | .0137 |
| **41** | -0.0109  (-0.0261 to 0.0042) | -0.0020  (-0.0171 to 0.0132) | 0.0090  (0.0082 to 0.0097) | -0.0108  (-0.0266 to 0.0049) | -0.0022  (-0.0179 to 0.0135) | 0.0086  (0.0079 to 0.0094) | -0.0004  (-0.0015 to 0.0007) | .7150 | .7497 |
| **42** | 0.0033  (-0.0094 to 0.0161) | -0.0012  (-0.0139 to 0.0116) | -0.0045  (-0.0052 to -0.0038) | -0.0212  (-0.0387 to -0.0036) | -0.0258  (-0.0434 to -0.0083) | -0.0047  (-0.0054 to -0.0040) | -0.0002  (-0.0013 to 0.0009) | .9440 | 1 |
| **43** | -0.0141  (-0.0356 to 0.0074) | -0.0152  (-0.0367 to 0.0064) | -0.0011  (-0.0020 to -0.0002) | -0.0117  (-0.0339 to 0.0105) | -0.0043  (-0.0265 to 0.0179) | 0.0074  (0.0067 to 0.0082) | 0.0085  (0.0074 to 0.0096) | < .0001 | < .0001 |
| **44** | 0.0040  (-0.0111 to 0.0191) | 0.0137  (-0.0014 to 0.0288) | 0.0098  (0.0091 to 0.0104) | 0.0061  (-0.0146 to 0.0267) | 0.0204  (-0.0003 to 0.0411) | 0.0144  (0.0137 to 0.0151) | 0.0046  (0.0036 to 0.0056) | < .0001 | < .0001 |
| **45** | 0.0065  (-0.0072 to 0.0203) | 0.0161  (0.0023 to 0.0298) | 0.0096  (0.0087 to 0.0105) | -0.0099  (-0.0404 to 0.0207) | -0.0031  (-0.0337 to 0.0274) | 0.0067  (0.0058 to 0.0077) | -0.0029  (-0.0043 to -0.0015) | .0033 | .0047 |
| **46** | -0.0064  (-0.0193 to 0.0065) | -0.0086  (-0.0215 to 0.0043) | -0.0022  (-0.0029 to -0.0015) | -0.0230  (-0.0484 to 0.0023) | -0.0222  (-0.0475 to 0.0032) | 0.0009  (-0.0002 to 0.0019) | 0.0031  (0.0020 to 0.0042) | < .0001 | 0.0001 |
| **47** | 0.0015  (-0.0179 to 0.0209) | 0.0023  (-0.0171 to 0.0217) | 0.0008  (0.0001 to 0.0016) | -0.0058  (-0.0181 to 0.0065) | 0.0019  (-0.0104 to 0.0142) | 0.0076  (0.0068 to 0.0085) | 0.0068  (0.0055 to 0.0081) | < .0001 | < .0001 |
| **48** | -0.0088  (-0.0216 to 0.0040) | -0.0033  (-0.0161 to 0.0095) | 0.0055  (0.0049 to 0.0061) | -0.0082  (-0.0294 to 0.0129) | 0  (-0.0211 to 0.0212) | 0.0083  (0.0075 to 0.0091) | 0.0028  (0.0018 to 0.0038) | < .0001 | < .0001 |

Values are mean (95% confidence interval)
